# Realizing the Potential of *Camelina sativa* as a Bioenergy Crop for a Changing Global Climate

**DOI:** 10.3390/plants11060772

**Published:** 2022-03-14

**Authors:** Dhurba Neupane, Richard H. Lohaus, Juan K. Q. Solomon, John C. Cushman

**Affiliations:** 1 MS330/Department of Biochemistry & Molecular Biology, University of Nevada, Reno, NV 89557, USA; dneupane@unr.edu (D.N.); rhlohaus25@gmail.com (R.H.L.); 2 Department of Agriculture, Veterinary & Rangeland Sciences, University of Nevada, Reno, NV 89557, USA; juansolomon@unr.edu

**Keywords:** *Camelina sativa*, semi-arid lands, biofuel feedstock, biodiesel, renewable diesel, crop breeding, transgenesis, genome editing

## Abstract

*Camelina sativa* (L.) Crantz. is an annual oilseed crop within the Brassicaceae family. *C. sativa* has been grown since as early as 4000 BCE. In recent years, *C. sativa* received increased attention as a climate-resilient oilseed, seed meal, and biofuel (biodiesel and renewable or green diesel) crop. This renewed interest is reflected in the rapid rise in the number of peer-reviewed publications (>2300) containing “camelina” from 1997 to 2021. An overview of the origins of this ancient crop and its genetic diversity and its yield potential under hot and dry growing conditions is provided. The major biotic barriers that limit *C. sativa* production are summarized, including weed control, insect pests, and fungal, bacterial, and viral pathogens. Ecosystem services provided by *C. sativa* are also discussed. The profiles of seed oil and fatty acid composition and the many uses of seed meal and oil are discussed, including food, fodder, fuel, industrial, and medical benefits. Lastly, we outline strategies for improving this important and versatile crop to enhance its production globally in the face of a rapidly changing climate using molecular breeding, rhizosphere microbiota, genetic engineering, and genome editing approaches.

## 1. Introduction

*Camelina sativa* (L.) Crantz., also known as false or wild flax, German sesame, gold-of-pleasure, or linseed dodder, is an allohexaploid (2n = 40) oilseed crop within the Brassicaceae [1,2,3,4,5,6]. Interest in *C. sativa* increased in recent years due to its adaptability to diverse environmental conditions, low requirements for water and nutrients, relatively strong resistance to insect pests and microbial diseases, and unique oil composition and characteristics suitable for the production of food and fodder, biofuels, and bio-based products [1,2,7,8]. These positive agronomic traits and environmental attributes, along with the recent development of methods for transgenesis [9,10,11,12,13] and CRISPR/Cas genome editing [14,15,16], triggered great interest in *C. sativa* as an industrial oilseed crop. The ongoing interest in *C. sativa* is documented by the large number of peer-reviewed publications from various databases retrieved when “camelina” was used as a search term, for example, in a query of the ScienceDirect (2309 publications from 1997–2021), Web of Science (1525 publications from 2000–2021) and Agricola (677 publications from 2000–2021) databases (reported on December 30, 2021). The large number of publications and data reported on *C. sativa* highlight the immense potential of this crop and the interest in genetic improvement to allow it to gain more widespread acceptance and economic viability. Most publications focus on the uses of *C. sativa* oil and meal, the composition of oil and fatty acids, its genetics and breeding, its physiology, and its production and agronomic management. Agronomic management refers to the practice of minimizing input factors such as fertilizer, irrigation, tillage, herbicides, fungicides, and insecticides to maximize crop yield outputs such as seed yield, oil content, and biodiesel production. Many reviews report on various aspects of its general use as an oilseed crop [2,4,17,18,19,20,21,22,23,24], and as a platform for the production of biofuels [25,26,27,28] and industrial lipids [1,6,8]. After providing some essential background, we summarize the present status of *C. sativa* research and identify areas for its future improvement with a particular emphasis on enhancing the climate resilience of this highly versatile crop.

## 2. Origin and Distribution

*Camelina sativa* is an ancient crop, known as early as 4000 BCE in Auvervier, Switzerland [29] with evidence of widespread cultivation throughout northern Europe from Southern Scandinavia [6,30] to central Asia (eastern Turkey), from 700–900 BCE [31] to the Iron Age (100 CE–250 BCE) [24]. Archaeological sites revealed evidence that *C. sativa* was cultivated for food and oil production in Scandinavia, Romania, and eastern Turkey during the late Stone Age and middle Bronze Age (1800 BCE) [32], with widespread availability during the late Bronze age (1200 BCE) [30]. *C. sativa* cultivation declined during the Medieval Age, but grew during the last century throughout northern, central and eastern Europe, the Balkans, and Russia and to some extent in North America [33]. However, much of its former cultivation all but disappeared, being replaced almost entirely by rapeseed (*Brassica napus* L.) [24].

*C. sativa* was likely introduced to the Americas as a weed in flax, thus giving rise to the name false flax [4]. Today, *C. sativa* is cultivated throughout the northern USA and southern Canada [34]. Within the USA, *C. sativa* is grown effectively in the Pacific Northwest, across the North and Central Plains into the Corn Belt region [35], and in the arid Southwest with irrigation [36,37]. Within Canada, *C. sativa* is grown widely from the western Prairie Provinces [38,39] to the eastern Maritime Provinces [40].

## 3. Genetic Diversity and Morphological Variation

*C. sativa* belongs to the tribe Camelineae within the Brassicaceae. The similarity between *C. sativa* and *Arabidopsis thaliana* [41,42] makes *A. thaliana* a useful reference for the development of genetic and genomic tools in *C. sativa* [34]. The genus *Camelina* has up to 11 species, revealing taxonomic dissimilarity with its center of diversity in Eurasia (Russia or Ukraine) [43,44,45]. Among 11 species, five species, namely *C. sativa*, *C. microcarpa*, *C. rumelica*, *C. alyssum*, and *C. hispida,* are found in Europe and three species, namely *C. sativa*, *C. microcarpa*, and *C. alyssum,* are found in the USA and Canada [11]. Among these species, only *C. sativa* and *C. microcarpa* are cultivated [11]. *C. sativa* is an allohexaploid species with 2n = 6x = 40 [34,46]. Other *Camelina* species such as *C. hispida* (Boiss.) Hedge (2n = 2x = 14), *C. neglecta* (2n = 2x = 12) [44], and *C. laxa* C.A. Mey. (2n = 2x= 12) [47] are diploid. Other species are polyploid, including *C. rumelica* Velen., tetraploid (2n = 4x = 26); *C. microcarpa* Andrz. ex DC with three cytotypes: diploid (2n = 2x = 12), tetraploid (2n = 4x = 26), and hexaploid (2n = 6x = 40) [48]; and *C. sativa*, hexaploid (2n = 2x= 40) [49,50]. Morphologically, *C. sativa* and *C. microcarpa* are very similar except for the smaller seed size of *C. microcarpa* [51]. This phenotypic similarity suggests that *C. sativa* could potentially be the domesticated form of *C. microcarpa* [43]. Greater variations in chromosome counts within species might arise from intraspecific ploidy variations or inaccurate records suggesting past taxonomic misidentification [52]. Investigations into the genome structure of 193 *Camelina* accessions revealed three subpopulations, with two represented by domesticated *C. sativa* accessions and one composed of *C. microcarpa* species that included a newly designated *C. neglecta* diploid (née *C. microcarpa*) species [53]. Recent molecular phylogenies derived from chloroplast genome sequencing of 84 individuals revealed low intragenic variation across the *Camelina* genus [54]. However, cytotypes and chromosome counts across 82 individuals confirmed that the tetraploid *C. microcarpa* (or *C. neglecta*-like) is the proposed maternal parent and the diploid *C. hispida* is the proposed paternal parent of *C. sativa* [53,54].

Resembling other genera within the *Brassicaceae*, *Camelina* species are dicotyledonous with high morphological plasticity. Biologically, *Camelina* species can be annual spring or biennial winter types [2], with some species requiring vernalization to induce flowering [55]. Most domesticated *C. sativa* are spring types, whereas most wild relatives are winter types. Winter types are well adapted as cover crops in double- or relay-cropping systems, with soybean (*Glycine max* L. Merr.) and other short-seasoned summer crops due to their early maturity in the Great Plains and the Upper Midwestern USA [3,35,56,57,58]. *C. sativa* typically has a short growing cycle of 85–120 days [4,5,22,50,59]. Growing degree day (GDD) requirements for *C. sativa* during its complete life cycle (with up to 75% ripe silicles) generally range from 1200 to 1300 °C with a base temperature of 5 °C [60]. Plant height ranges from 30 to 120 cm depending upon cultivars, growing season, and amount of nitrogen fertilizer used [4,36,59,61,62]. Stems are either hairy or smooth, are branched, and become lignified when mature. Leaves are arrow-shaped and pointed, approximately 5–8 cm long with smooth to undulated edges. Flowers are 5–7 mm in diameter and are mostly autogamous [49]. Flowers are pale yellow in color and arranged within inflorescences, called raceme. The silicles are 5–14 mm long, slightly flattened, and pear-shaped, containing 8–15 golden to brown colored seeds at maturity. *C. sativa* seeds are very small, with 1000 seed weight varying between 0.8 to 1.8 g, depending upon the cultivar and growing environments during seed growth and development [4,22,24,63,64]. *C. sativa* has a deep taproot system, varying with soil type and growing conditions, which is thought to improve nutrient scavenging. The use of *C. sativa* as a cover crop can reduce nutrient run-off, particularly for winter annual cultivars [2]. *C. sativa* can also reduce soil compaction and improve infiltration capacity, similar to other species within the *Brassicaceae* [2].

## 4. *C. sativa* Yield Potential under Hot and Dry Conditions

Global climate change is related to increasing surface aridification and the increasing duration and frequency of droughts in many regions of the world [65,66]. Interest in *C. sativa* as a low-input crop for use on marginal lands has grown in recent years [50,67,68,69,70,71]. Thus, recent studies investigated *C. sativa* grown under semi-arid conditions with limited water inputs, with the goal of assessing its performance under reduced water and fertilizer inputs [36,37,59,62,72].

**Figure 1:**
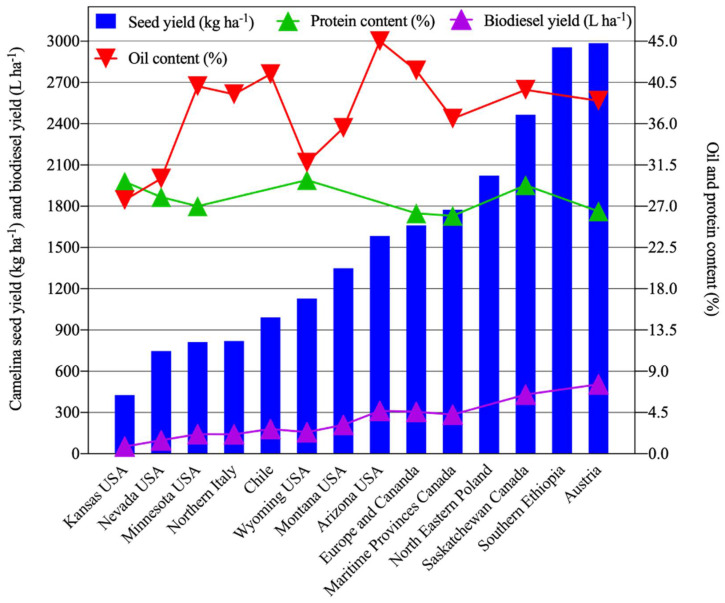
Average seed yield (kg ha^−1^), oil (%), and protein content (%) of *C. sativa* grown at various locations across the world.

Many studies were conducted across semi-arid or arid regions of the globe with distinct mean annual precipitation levels to compare seed yield (kg ha^−^^1^), oil content (%), protein content (%), and biodiesel production (L ha^−^^1^) (Figure 1, Table 1). Differences in cultivars (genotypes), water availability, environment, physical and chemical characteristics of soils, and management practices such as irrigation, nitrogen application, sowing date, seeding methods and rates, clearly impact overall *C. sativa* productivity. Among these studies, the overall mean seed yield of *C. sativa* was 1410 kg ha^−^^1^ (Figure 1, Table 1). The highest reported seed yield ranges were attained in Austria (2419–3625 kg ha^−^^1^) [73] and in southern Ethiopia (2795–3200 kg ha^−^^1^) [74]. The lowest *C. sativa* seed yield ranges were reported by studies performed in Kansas [75] and Nevada, USA [36,37,59,62]. Low seed yield of *C. sativa* may occur due to drought and high temperatures during the flowering and pod filling stage [61,76], as well as poor soil quality [77]. Consistent with these findings, environmental factors (e.g., temperature and rainfall) were responsible for approximately 73% of the variation in seed yield in north-eastern Poland [72]. In contrast, only ~6% of the variation in seed yield was due to genetic factors [72]. Mild weather conditions, along with moderate in-season precipitation, favor higher seed yields [35,39]. Additionally, high *C. sativa* seed yields were associated with adequate moisture and mild temperatures during seed filling, which is critical for the production and transport of sugars from source to sink tissues [3,77,78]. In contrast, low seed yields were observed under conditions of hot temperature and low precipitation [79,80]. Studies showed an increase in seed yields ranging from 425 to 2867 kg ha^−^^1^ as a result of increasing amounts of applied irrigation ranging from 187 to 536 mm [36,69,70,81]. Seasonal water requirements range from 332–490 mm for *C. sativa* based upon cumulative evapotranspiration (ET) estimates [82,83,84]. Notably, *C. sativa* might serve as a reliable alternative to Canola in locations where seasonal water supply is less than 250 mm [85]. In addition to increased water inputs, increasing nitrogen fertilizer application can increase seed yield. Seed yields of 1800 kg ha^−1^ can be attained with 150 kg N ha^−1^ and water inputs in the range of 320–376 mm [86]. Similar estimates for optimal N input were reported for rainfed field settings [87,88]. The use of organic fertilizers during the production of *C. sativa* for animal feed reduces its environmental impact [89].

Another important measure of productivity for any oilseed crop is oil and protein production of the seed meal. For *C. sativa*, oil and protein content varied widely across various parts of the world, with overall mean seed oil and protein contents of ~36% and ~28%, respectively (Figure 1, Table 1). Highest seed oil contents were reported in the range of 39.8–45.7% in Chile [61], followed by 45% in Arizona, USA [84], and 38–43% in western Canada [39]. The lowest seed oil content, ranging from 25.7 to 31.8%, was observed in Nevada, USA [37]. Similarly to seed yield, oil yield is strongly influenced by environmental factors [64,90]. For example, oil content variation was chiefly associated with mean air temperature and water availability during seed development [61].

**Table 1:** *C. sativa* seed yield, oil, and protein content reported across various regions of the world.

Locations	Seed Yield (kg ha^−1^)		Seed Oil Content (%) ^1^	Seed Meal Protein Content (%) ^2^	Biodiesel Yield (L ha^−1^) ^3^	Major Sources of Variation	Reference
	Mean	Range	Range	Range	Mean		
Austria	2986	2419–3625	37.0–40.0	25.0–27.9	505	Nitrogen and sulfur rates	[73]
Southern Ethiopia	2956	2795–3200	-	-	-	Seeding rates and nitrogen fertilizer	[74]
Saskatchewan, Canada	2466	2184–2747	38 to 43 (39.7)	27 to 32 (29.3)	430	Genotypes and environment	[39]
North–Eastern Poland	2023	1700–2210	-	-	-	Genotypes	[72]
Maritime Provinces of Eastern Canada	1775	1638–1911	33.8–39.0 (36.6)	25.0–26.8 (26.0)	285	Breeding lines, nitrogen and sulfur rates	[87]
Europe and Canada	1660	1100–2700	(41.8)	(26.2)	305	Genotypes and environment	[77]
Arizona, USA	1583	1527–1638	(45)	-	313	Nitrogen rates, water use and irrigation scheduling	[84]
Montana USA	1349	546–2942	33.5–37.6 (36.0)	-	211	Cultivars and locations	[78]
Wyoming, USA	1129	832–1643	31.1–32.4	29.3–30.4	157	Nitrogen and sulfur rates	[91]
Chile	991	387–2314	39.8–45.7 (41.41)	-	180	Cultivars, planting dates, and locations	[61]
Northern Italy	820	600–940	(39.2)		141	Low input and growing seasons	[71]
Nevada, USA	899	770–1013	31.8–33.3 (32.4)	27.1–28.1 (27.5)	128	Cultivars and irrigation rates	[36]
	784	534–1010	31.8–32.6 (32.2)	26.2–30.4 (28.4)	111	Cultivars, nitrogen sources and rates	[59]
	735	34–1921	26.6–30.8	-	69.7	Cultivars and year	[37]
	570	130–921	28.4–29.5	-	72	cultivars, sowing date, and methods	[62]
Minnesota, USA	812	650–944	39.4–40.7 (40.1)	26.2–27.9 (27.0)	143	Genotypes and seeding rate	[3]
Kansas, USA	427	317–503	27–29 (27.7)	29–30 (29.7)	52	Cultivar and planting date	[75]
**Overall mean**	**1410**		**36.0**	**27.8**	**208.4**		

^1^ Oil and ^2^ protein content inside brackets indicate the average oil and protein content (%). ^3^ Biodiesel yield was estimated by multiplying seed yield (kg ha^−1^) and oil content (%) using the volumetric conversion factor of 1 kg ha^−1^ to 0.439 L ha^−1^ [92].

Compared with seed oil values, protein content generally varied less widely relative to the location of production across the globe, with the lowest and highest ranges of 25.0–26.8% and 27–32%, respectively, reported in Canada, with an overall mean of 27.8% (Figure 1, Table 1). However, more wide-ranging protein contents (23–47%) of *C. sativa* seeds were reported depending upon local field conditions [60,77]. High air temperatures during flowering and seed filling are generally associated with low oil contents but high protein contents within *C. sativa* seed [62]. Furthermore, increased N application rates can increase protein contents, with an associated decrease in oil contents [73,87,93,94]. Such increased nitrogen applications are known to increase protein at the expense of fatty acid synthesis due to the competition for carbon skeleton during carbohydrate metabolism [95].

A major use of *C. sativa* seed oil is biodiesel production. Biodiesel production, which is closely linked with overall oil production, varied widely with a mean of 208 L ha^−^^1^ across the globe (Figure 1, Table 1). The highest biodiesel yield (505 L ha^−^^1^) was reported in Austria [73], followed by western Canada (430 L ha^−^^1^) [39]. A multi-location study performed in Europe and Canada reported a mean biodiesel yield of 305 L ha^−^^1^ [77]. The lowest biodiesel yield (52 L ha^−^^1^) was reported in Kansas, USA [75]. The wide variation in biodiesel production is likely accounted for by differences in temperature and water availability, including ambient precipitation, length of growing season, and managerial factors such as fertilizer application rates and irrigation practices.

## 5. Biotic Production Constraints of *C. sativa*

Weeds are a major constraint to *C. sativa* production (Berti et al., 2016). *C. sativa* is considered to be very competitive against weeds with good stand establishment [22,96], due to its ability to produce and release secondary metabolites that prevent the growth of seedlings of neighboring plants [97,98,99]. However, the competitiveness of *C. sativa* for sunlight, nutrients, and water can be challenged by some weed species such as cheatgrass (*Bromus tectorum*), green foxtail (*Setaria viridis* L.), and Russian thistle (*Salsola kali* L.), among others [2,5,100,101]. Similarly, perennial broadleaf weeds such as field bindweed (*Convolvulus arvensis* L.), Canada thistle (*Cirsium arvense* L. Scop.), and skeleton weed (*Chondrilla juncea* L.) can also present challenges for *C. sativa*. Notably, *C. sativa* can exhibit susceptibility to dodder (*Cuscuta* spp.), a parasitic weed of many crops (D. Neupane, personal observation). *C. sativa* does not hybridize efficiently with cultivated *Brassica* species or wild *Brassica* species within the tribe *Camelineae* [102]. However, *C. sativa* does exhibit the ability to outcross with common *Brassica* weed species, such as North American weeds *C. alyssum* and *C. microcarpa* [103]. Fortunately, *C. sativa* is less competitive and shows lower invasive potential than Canola [104]. 

### 5.1. Weed Control

Both chemical and non-chemical approaches for weed control are available for *C. sativa* production systems. For chemical control, Sethoxydim (Poast^®^) (2-[(E)-N-ethoxy-C-propylcarbonimidoyl]-5-(2-ethylsulfanylpropyl)-3-hydroxycyclohex-2-en-1-one), a post-emergent and selective grass herbicide, which belongs to the cyclohexanone group, is currently the only registered herbicide for *C. sativa* in the USA [59,62,105,106]. Tepraloxydim (2-[(E)-N-[(E)-3-chloroprop-2-enoxy]-C-ethylcarbonimidoyl]-3-hydroxy-5-(oxan-4-yl)cyclohex-2-en-1-one) was also used to control post-emergent grass weeds [61]. Similarly, Clethodim (2-[(E)-N-[(E)-3-chloroprop-2-enoxy]-C-ethylcarbonimidoyl]-5-(2-ethylsulfanylpropyl)-3-hydroxycyclohex-2-en-1-one), which is a related post-emergent cyclohexanone herbicide, was successfully used for grassy weed control for *C. sativa* [36]. Quizalofop (2-[4-(6-chloroquinoxalin-2-yl)oxyphenoxy]propanoic acid), which is a selective, postemergence phenoxy herbicide registered in Canada and other parts of the world, was also used to control annual and perennial grass weeds of *C. sativa* [2,5,107].

In contrast to selective grass herbicides, *C. sativa* withstands very few broadleaf herbicides, and few have been approved for use on *C. sativa* in the USA [2,5,22,107,108]. Dinitroaniline pre-emergent herbicides, such as Trifluralin (2,6-dinitro-N,N-dipropyl-4-(trifluoromethyl)aniline), are widely used in the USA for early season weed control. Pre-emergent herbicides, including dimethenamid-P (2-chloro-N-(2,4-dimethylthiophen-3-yl)-N-[(2S)-1-methoxypropan-2-yl]acetamide), pendimethalin (3,4-dimethyl-2,6-dinitro-N-pentan-3-ylaniline), pyroxasulfone (3-[[5-(difluoromethoxy)-1-methyl-3-(trifluoromethyl)pyrazol-4-yl]methylsulfonyl]-5,5-dimethyl-4H-1,2-oxazole), quinclorac (3,7-dichloroquinoline-8-carboxylic acid), and S-metolachlor (2-chloro-N-(2-ethyl-6-methylphenyl)-N-[(2S)-1-methoxypropan-2-yl]acetamide), were tested at three different concentrations with *C. sativa* to examine weed control and injury to the crop [109]. Pendimethalin and S-metolachlor caused some injury, but did not lower *C. sativa* seed yields. Dimethenamid-P, used at a low rate, did not affect plant populations and seed yield; however, when applied at a high rate, it caused 60% injury with a 31% reduction in seed yield [109,110]. Quinclorac did not significantly injure the crop and was considered the safest of all the herbicides tested [109]. *C. sativa* mutants with resistance to acetolactate synthase (ALS) inhibitors were developed, displaying increased resistance to imazethapyr (5-ethyl-2-(4-methyl-5-oxo-4-propan-2-yl-1H-imidazol-2-yl)pyridine-3-carboxylic acid), sulfosulfuron (1-(4,6-dimethoxypyrimidin-2-yl)-3-(2-ethylsulfonylimidazo[1,2-a]pyridin-3-yl)sulfonylurea), and flucarbazone (3-methoxy-4-methyl-5-oxo-N-[2-(trifluoromethoxy)phenyl]sulfonyl-1,2,4-triazole-1-carboxamide) [110].

Several non-chemical strategies for weed control were shown to be effective for *C. sativa* [96]. Mechanical weeding is a well-established and useful approach for small scale production systems (e.g., research plots). For large-scale production, early season planting that promotes stand establishment is one of the most effective strategies for overcoming weed competition in *C. sativa* fields [111]. Furthermore, increasing sowing rates can effectively suppress weeds in *C. sativa* plots, likely through increased utilization of resources by the crop at the expense of weeds [96]. Mixed cropping of *C. sativa* grain with peas (*Pisum sativum*) (or barley (*Hordeum vulgare*)) afforded significant weed suppression and acted as a smother crop and weed antagonist, leading to enhanced *C. sativa* seed yields [96,112].

Use of *C. sativa* as a winter cover crop suppressed weed abundance while increasing cash crop yields [113]. Including *C. sativa* in crop rotations for winter cereals can suppress winter weed populations and increase grain yields in semi-arid Mediterranean climates [114]. *C. sativa* was also used in innovative double- and relay-cropping systems with forage sorghum (*Sorghum bicolor*), maize (*Zea mays*), and soybean (*Glycine max*) with an increased potential for biofuel and energy feedstock production [115]. When *C. sativa* was intersown as a cover crop with maize or soybean, establishment and winter survival rates increased, with the greatest success being achieved with soybean [116]. *C. sativa* was also used as an intercrop with *Jatropha integerrima* in semi-arid regions of India with seed production improvements observed when grown alternatively with leguminous fodder crops [117]. Further exploration of the potential allelopathic effects of *C. sativa* in intercropping systems for weed suppression is needed [99].

### 5.2. Insect Pests

Insects can limit *C. sativa* production, but these generally present lower production barriers than weeds. *C. sativa* is known to be relatively resistant to insect predation and infestation. *C. sativa* shows resistance to several common insect pests of Canola [118,119]. For example, *C. sativa* shows high resistance to flea beetles (*Phyllotreta* spp.), a common insect pest of *B. rapa*, *B. napus*, and *B. juncea* [39,49,120,121]. This resistance is thought to be due to the presence of repellents or the absence of stimulatory volatile phytochemicals [122]. More recent studies confirmed that the presence of quercetin glycosides in *C. sativa* leaves is likely responsible for flea beetle resistance [123]. In addition to flea beetles, *C. sativa* was shown to be a poor host for the diamondback moth (*Plutella xylostella* L.) and root maggots (*Delia* spp.) [124]. Feeding damage by the Bertha armyworm (*Mamestra configurata* Walker) on *C. sativa* was consistent with Canola, but larvae and pupae weighed less when reared on *C. sativa* leaves [124]. Leafhopper (*Macrosteles quadrilineatus* Forbes) did not cause damage to *C. sativa*; however, this insect can transmit aster yellows phytoplasma, which can result in chronic, systemic disease resulting in chlorosis, phyllody, and virescence, malformed seeds, and reduced seed set [124]. *C. sativa* also appeared to show resistance against the cabbage seedpod weevil (Ceutorhyncus obstrictus (Marsh.) [125]. However, stem feeding below the soil by several weevil species (e.g., *Ceutorhynchus cyanipennis* and *C. americanus*) was associated with stand failures for fall-planted *C. sativa* [126]. Although aphids can colonize *C. sativa* under field conditions, they are not known to cause significant economic damage; however, such colonization can create a reservoir for different aphid species that can negatively impact other crops in the rotation [127].

### 5.3. Fungal, Bacterial, and Viral Pathogens

Similar to its apparent resistance to many insect pests, *C. sativa* shows good resistance to a variety of microbial pathogens that typically target crops within the *Brassicaceae* [18,128]. For example, *C. sativa* showed strong resistance to blackspot fungal disease caused by *Alternaria brassicae* (Berk.) Sacc. [129,130,131]. Such resistance is associated with the relative expression of defense-related genes [132]. Resistance to blackspot disease caused by *Alternaria brassicicola* [(Schw.) Wiltsh] varies among *C. sativa* genebank accessions [133]. However, such resistance can be transferred to hybrids of *C. sativa* and rapid-cycling *Brassica oleracea* in an effort to confer resistance of this disease to *Brassica* vegetable crops [134,135]. Resistance to the blackspot pathogen was associated with the production of phytoalexins, specifically to camalexin, an antimicrobial phytoalexin [129,131,134,136]. Resistance was also correlated with the higher expression of chitinase gene family members in *C. sativa* relative to *B. juncea* [137]. *C. sativa* showed strong resistance to blackleg fungal disease [39] assessed using 80 different isolates of *Leptosphaeria maculans* (Desmaz.) [138]. However, for those *C. sativa* genotypes that are susceptible to blackleg disease, the fungicides metoconazole and a mixture of tebuconazole and triadimenol provided very effective control against the fungus [61].

*C. sativa* genotypes show various, but low, degrees of susceptibility to common *Brassicaceae* diseases, such as stem rot (*Sclerotinia sclerotiorum* (Lib.) de Bary), brown girdling root rot (*Fusarium* spp. and *R. solani*), downy mildew (*Peronospora sparsa*, *P. parasitica*), powdery mildew (*Erysiphe* sp.), grey mold (*Botrytis cinerea*), sore shin and damping-off disease (*Rhizoctonia solani*), Verticillium wilt (*Verticillium dahliae*), and white rust (*Albugo candida*) [18,128,139,140]. Some *C. sativa* genotypes are susceptible to downy mildew (*Hyaloperonospora camelinae*) [141]. Thirty diverse *C. sativa* genotypes showed varying degrees of resistance to *S. sclerotiorum* [142]. Resistance to *S. sclerotiorum* was associated with cell wall strengthening due to monolignol biosynthesis in *C. sativa* [143]. However, while camalexin production was induced by S. *sclerotiorum* inoculation, the relative degree of disease resistance was not correlated with levels of camalexin production, suggesting that other antimicrobial activities might be responsible for the observed disease resistance [142]. *C. sativa* also shows resistance to the soil-born fungus Fusarium *virguliforme* [144]. However, *C. sativa* is susceptible to other fungal diseases common to the *Brassicaceae,* such as damping-off (caused by *Rhizoctonia solani*), clubroot (*Plasmodiophora brassicae* Woronin.), and white rust (*Albugo candida* Pers. Kuntze) [128]. While susceptible, *C. sativa* showed greater resistance to *Rhizoctonia solani* than *B. napus* did, due to the presence of the phytoalexins camalexin and methoxycamelexin and two additional antimicrobial compounds in its roots [145].

*C. sativa* is susceptible to some bacterial diseases, such as bacterial blight (*Pseudomonas syringae* pv. spec.), phytoplasma disease, which was reported for *C. sativa* grown in Germany and Canada [139,140,146], and aster yellows diseases [124]. *C. sativa* also shows susceptibility to the bacterial pathogen *Xanthomonas campestris* Dowson pv. campestris, which causes black rot of *Brassica* crops globally [133].

Lastly, although relatively less well studied for viral diseases, *C. sativa* is susceptible to the Turnip crinkle virus and Turnip rosette virus [147], which are viral diseases transmitted by flea beetles via infested seed [2,148]. The beet western yellows mosaic virus reportedly caused up to 34% yield reductions in *C. sativa* [149]. Aphid (*Myzus persicae*) vector behavior and host palatability are key factors in the transmission of the Turnip yellows virus in *C. sativa* [150].

## 6. Ecosystem Services Provided by *C. sativa*

In addition to its role in crop rotations, *C. sativa* may be used to provide a range of ecosystem services, including prevention of soil loss and erosion, habitat for pollinators, and phytoremediation. Regrowth of hardy winter *C. sativa* can help to limit soil erosion and nutrient run off in the early spring [35]. In general, the use of *C. sativa* as a cover crop, with its deep tap root, is expected to benefit soil structure, promote nutrient recycling, and enhance nutrient scavenging [2]. Although *C. sativa* produces relatively small amounts of biomass compared with larger crops, these crop residues can promote soil water absorption capacity, which is particularly important in areas with dry soils [37]. Nonetheless, no-till and low-till cultivation methods are recommended for *C. sativa* to avoid or reduce soil erosion [111]. Both winter and spring *C. sativa* can provide pollinators with early season nectar and pollen for honey bees and other pollinators, as its flowers open long before most other crops grown in many regions [151,152]. Furthermore, *C. sativa* nectar sugar produced throughout anthesis can exceed that of pennycress (*Thlaspi arvense*) or Canola (*Brassica napus*) [151]. Cocultivation of winter *C. sativa* with Persian clover was found to decrease the heavy metal (i.e., copper, lead, nickel, and zinc) content of soils [153]. Lastly, *C. sativa* can be used similarly to other cover crops, including those within the *Brassicaceae*, to promote atmospheric carbon sequestration, suppress weeds, provide erosion control, protect ground water quality, promote organic matter carbon and nitrogen accumulation in soils, and suppress the accumulation of fungal pathogens, particularly saprophytic fungal species, and nematodes [154,155].

## 7. Fatty Acid Synthesis and Seed Oil Profiling in *C. sativa*

*C. sativa* seed oil content varies from approximately 27 to 46% and has a high omega-3 fatty acid content [26,39,49,64]. *C. sativa* seed oil contains 90% unsaturated fatty acids (~60% polyunsaturated fatty acids (PUFAs), 30% monounsaturated fatty acids (MUFAs)), and 10% saturated fatty acids (SFAs). In *C. sativa* oil, palmitic (C16:0), oleic (C18:1), linoleic (C18:2), and α-linolenic (C18:3) acids are the predominant fatty acids [156]. *C. sativa* seed oil is unique in its oil composition and chemical characteristics because polyunsaturated α-linolenic acid (C18:3) is the major fatty acid, and the concentration of erucic acid (C22:1) is present in a comparatively low amount for a *Brassicaceae* species, whereas eicosenoic acid (C20:1) is produced instead as the major long-chain fatty acid [6].

The mean fatty acid composition of *C. sativa* oil includes α-linolenic acid (34.4%), linoleic acid (18.3%), oleic acid (14.7%), gadoleic acid (C20:1, 14.0%), and palmitic acid (5.8%) in descending order of relative abundance (Table 2). The mean erucic acid content was 2.9%, which is below the maximum threshold value allowed for biodiesel (5%) [157], and just above the 2% threshold required for food-grade oil in the USA [3]. Other fatty acids such as stearic acid (C18:0, 2.6%), docosaenoic acid (C22:0), nervonic acid (C24:1), ecosadienoic acid (C20:2), dihomo-gamma linolenic acid (C20:3), and docosadienoic acid (C22:3) are also present in trace amounts. The relative content of the major fatty acids varied widely depending on the study. For example, α-linolenic acid ranged from 22.8–38.4% [158] to 41% [157]. Linoleic acid ranged from 12.8–20.6% [159,160,161]. Oleic acid ranged from 6.9–22.1% [158]. The ranges of other fatty acids, such as gadoleic acid, palmitic acid, and erucic acid, are summarized (Table 2). The wide variations in fatty acid profiles of *C. sativa* likely arise from differences in genotypes, agronomic practices, growing environment (e.g., soil quality), weather and climatic conditions [2,90,162,163].

The biosynthesis of triacylglyceride (TAG) in *C. sativa* occurs in the plastid via a Type II fatty acid synthase complex [169]. In most oilseed crops, the fatty acid chain elongates to 16 or 18 carbons in length [162]. Oleic acid (C18:1) (MUFA) is synthesized in the plastid from stearic acid (SFA) through steroyl-acyl carrier protein desaturase (SAD). Further desaturation of oleic acid to linoleic acid (C18:2) is catalyzed by fatty acid desaturase-2 (FAD2), a key enzyme responsible for the biosynthesis of PUFA in non-photosynthetic tissues, for example, the roots and developing seeds of oilseed plants in the ER and FAD 6 (plastidial enzyme) in the plastid [170,171]. α-linolenic acid (C18:3) synthesis from linoleic acid is catalyzed by FAD3 (microsomal enzyme) in the ER and FAD7/FAD8 (plastidial enzymes) in the plastid [172]. Fatty acid composition can change during seed development and varies with *C. sativa* accession. Fatty acid production can be affected by location due to climactic differences [157,173]. In oilseed crops, α-linolenic acid content differs depending on the temperature conditions during seed development. For example, at temperatures greater than 25 °C, the synthesis of α-linolenic acid declines because the activity of FAD3 decreases [2], whereas the synthesis of oleic and α-linoleic acid increases [174,175]. Similarly, biosynthesis of α-linolenic acid declined in non-irrigated compared with irrigated *C. sativa*, likely due to the higher temperatures of the non-irrigated plants [81]. Additionally, increasing temperatures from 15 °C to 35 °C reduced the expression of the FAD2 gene, suggesting a reduction in the synthesis of linoleic acid from oleic acid. A shorter duration of grain filling and a greater number of days above 25 °C resulted in a decline in linoleic acid content [176]. More recently, water-deficit stress was shown to increase oleic and linoleic acid content in *C. sativa* seeds, whereas linolenic acid content decreases, being dependent upon genotype, as one accession showed an increase in a-linolenic content following drought treatment [177]. Importantly, various environmental stress factors including heat, drought, salinity, high light, low oxygen, and high nitrogen can lead to decreases in overall seed oil content and changes in fatty acid composition [162]. Thus, an important consideration for improving overall seed oil yield is the need to improve the abiotic stress tolerance of *C. sativa*, particularly in the face of increasingly hot and dry conditions brought about by global warming.

## 8. Uses of *C. sativa*

**Figure 2:**
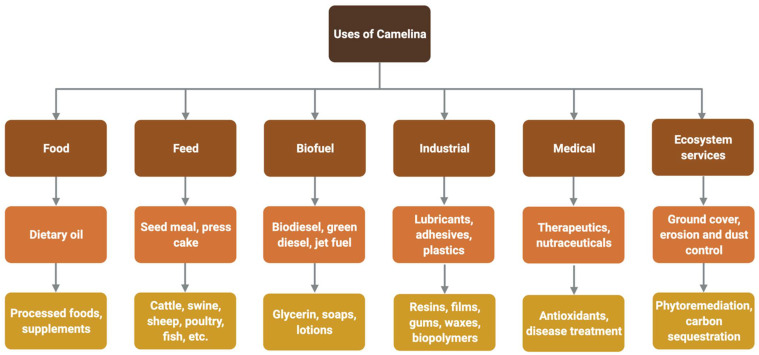
Diverse uses of *C. sativa* adapted from Chaturvedi et al., 2017 [17]. Created with BioRender.com.

*C. sativa* is used in a wide array of products and applications ranging from industrial, biomedical and nutraceutical products to animal feed, erosion control as ground cover, phytoremediation and carbon sequestration. *C. sativa* is also used in processed foods for human consumption. *C. sativa* is widely used as a biofuel, including biodiesel, green diesel, and renewable jet fuel. Derived products include glycerin, soaps and lotions (Figure 2).

### 8.1. Food and Food Products for Humans

*C. sativa* cooking or salad oil has potential human health benefits due to its high levels of PUFAs, omega-3-fatty acids, and antioxidants [156,178]. The consumption of *C. sativa* oil can reduce blood serum cholesterol levels [179] and improve serum lipid profiles [180,181]. Furthermore, *C. sativa* oil consumption can attenuate inflammation in peripheral blood mononuclear cells [182]. Such metabolic changes are thought to not only protect against cardiovascular risk factors, but also improve mental health in patients suffering from non-alcoholic fatty liver disease [183]. *C. sativa* oil was also used in folk medicine to treat burns and wounds to the skin and eyes [184,185]. The high levels of tocopherols, phytosterols, and carotenoids in *C. sativa* oil protect it from oxidation, imparting extended shelf life [185,186]. *C. sativa* oil displays antioxidant activities similar to those found in sunflower oil, which can effectively limit oxidation in food products, such as salad dressings and mayonnaise [156,187]. For example, *C. sativa* oil and rapeseed meal prevented the oxidation of lipids and proteins in cooked pork patties [188].

### 8.2. Feed for Animal Nutrition

Following oil extraction, *C. sativa* seed meal is toasted, dried, and cooled ready to use as animal feed for cattle, dairy cows, sheep, swine, and poultry, as well as for aquaculture of various fish stocks [2,189]. *C. sativa* seed and seed meal are generally considered useful and beneficial as animal feed in limited quantities. *C. sativa* seed displays a balanced profile of essential and non-essential amino acids [159,190].

#### 8.2.1. Beef Cattle

The exceptionally high level of omega-3 fatty acids, particularly α-linolenic acid (~32–40% of total oil content), protein (40%), oil (10–15%), fiber (0–15%), and phytate (1–6%), combined with relatively low glucosinolate levels (20–44 mmoles kg^−^^1^ dry weight), in *C. sativa* seed meal make it a suitable feed for animals [2,21,159,160,191,192]. *C. sativa* seed meal is generally comparable to Canola meal in terms of its amino acid profile, crude protein content, and digestibility, and was demonstrated to be a valuable feedstuff for ruminants [193,194,195]. However, some researchers report lower degradability and palatability of *C. sativa* seed meal compared with soybean or Canola meal [196,197]. Supplementation of the diet of beef cattle with *C. sativa* seed meal resulted in reduced forage and dry matter intake, but gave rise to greater serum PUFA concentrations [198]. In a related study, addition of 10% *C. sativa* seed meal on a dry weight basis did not affect dry matter intake and improved digestion of rumen organic matter, but did reduce body weights of dairy heifers [199]. Other studies report no negative effects on animal performance when *C. sativa* seed meal comprised up to 10% of the total diet [193]. *C. sativa* seed meal is highly degradable and shows comparable total digestibility and protein absorption to other cattle feed supplements [200]. Reduced intake or weight loss might be the result of the reduced palatability of *C. sativa* seed meal, as it contains anti-nutritive compounds, such as erucic acid, sinapine, and glucosinolates [196,201]. Due to these reasons, the U.S. Food and Drug Administration (FDA) permits only a maximum of 10% *C. sativa* seed meal in rations for livestock [202]. However, the sinapine content of *C. sativa* seed meal is lower than other *Brassica* species, and thus, only the glucosinolate content is relevant when assessing palatability [192]. Glucosinate content varies widely among accessions, and thus could be reduced through conventional breeding [192,203,204]. A high erucic acid content in seed meal can lead to fat deposition and reduced contractibility of the heart muscle and is thus limited to a maximum of <2% [205]. The erucic content in *C. sativa* seed meal ranges from 1.8–4.8% in the USDA National Genetic Resources Program collection [161]. Thus, similar to low glucosinolate accessions, accessions with low erucic acid content, such as *C. sativa* accession PI 650141, could be used for conventional breeding efforts or genome-editing approaches [161].

#### 8.2.2. Dairy Cows

*C. sativa* meal has a reasonably high level of histidine content, which is a good supplement for silage and grain-fed lactating cows [196]. Inclusion of seed or seed meal in the diet of dairy cows decreased dry matter intake, but did not have a significant effect on milk production [206]. However, overall milk protein and fat yield declined and resulted in a modified fatty acid profile with increased MUFAs and PUFAs, with resultant increases in butter spreadability [206]. Addition of *C. sativa* seed meal or oil to dairy cow diets did not alter dry matter intake, digestibility, or milk yield, but resulted in decreased saturated fatty acids and increased milk MUFA and PUFA content [207]. In a related study, supplementation of up to 6% *C. sativa* oil decreased milk yields and saturated fatty acid content while increasing unsaturated fatty acid content. This study concluded that 2% *C. sativa* oil could be used for commercial production without major adverse effects on animal performance [208].

#### 8.2.3. Sheep and Goat

Inclusion of 10–20% *C. sativa* seed meal in the diet of sheep increased the content of linoleic, oleic, and α-linolenic acid content in lamb muscle [209] and reduced blood triglycerides and glucose content, while increasing insulin levels [210]. Inclusion of *C. sativa* seed meal in sheep diets improved the total omega-3 fatty acid and the ratio of omega-6/omega-3 fatty acids in lamb and yearling meat, but resulted in reduced vitamin E content, which negatively impacted color and oxidative stability upon storage [211].

Supplementation of an ewe’s diet with 3–6% of *C. sativa* seed meal (or seed) in dietary dry matter increased the mono- and polyunsaturated fatty acid content of milk, resulting in milk with lower atherogenic and thrombogenic indices [212,213]. Similarly, inclusion of *C. sativa* seed meal in the diet of dairy goats increased total polyunsaturated fatty acids and decreased total saturated fatty acids in milk and kefir produced from the milk [214]. Inclusion of 10–20% *C. sativa* seed meal in the diet of dairy sheep altered the content and aroma of volatile compounds, primarily fatty acids, in raw and pasteurized ewe’s milk with an overall loss of dairy or freshness aroma [215]. Sheep or goat milk with increased fatty acid content resulted in increased fermentation time and alterations in the resulting kefir, and altered its aroma [216]. Additional studies validated these effects and showed that supplementation of sheep diets with varying amounts of *C. sativa* seed meal increased the mono- and/or poly-unsaturated fatty acids content and oxidative stability of the milk, resulting in a healthier milk for human consumption [217,218,219,220].

#### 8.2.4. Swine

After cold or expeller pressing, a comparatively high amount of oil (100–150 g kg^−^^1^) remains in the meal of *C. sativa*, which provides a potential energy source for swine diets [2]. Inclusion of 5–10% *C. sativa* seed meal in swine diets resulted in increased plasma omega-3 fatty acids and reduced plasma omega-6 fatty acids in blood plasma, while reducing serum triglyceride levels [221]. During the finishing–fattening process, supplementation of the swine diet with *C. sativa* oil increased α-linolenic acid content and reduced cholesterol content in the meat, thereby improving meat quality [222], and increased n-3 polyunsaturated fatty acid and reduced cholesterol and triglyceride of the plasma, implicating improved animal health [223]. Compared with sunflower seed meal, the inclusion of 12% *C. sativa* seed meal in the swine diet did not affect growth performance, but elevated the expression of some antioxidant defense system components in the spleen [224]. Digestibility of *C. sativa* seed meal in swine was comparable to that of Canola meal [225]. However, the addition of 20% *C. sativa* seed meal lowered digestibility coefficients for amino acids and crude protein, which limits its utilization in the swine diet [226]. Inclusion of 12% *C. sativa* seed meal increased α-linolenic acid content in the heart and brain with little alteration in performance [227]. Inclusion of *C. sativa* seed meal in the diets of weaned piglets at up to 18% increased n-3 fatty acids in carcass fat depots, but did not elicit clinical signs of toxicity, although it did result in lower average daily feed intake and weight gain than controls, probably due to an aversion to the taste of the *C. sativa* seed meal [228].

Supplementation of *C. sativa* seed meal improved feed efficiency and liver weight in 28-day-old weaned piglets, but also increased the expression of selected liver enzymes [229]. Comparison of the effects of the addition of 5%, 10%, or 15% *C. sativa* seed meal to the diet of growing–finishing pigs showed that the two higher percentages reduced average daily weight gain and marketing weight, but no negative growth performance or carcass traits were observed at 5% addition to a corn–soybean meal-based diet [230]. Incorporation of up to 30% *C. sativa* seed meal in a mixed corn/soybean meal diet for growing swine did not alter the digestible, metabolizable, or net energy, suggesting that dietary glucosinolates from *C. sativa* seed meal did not affect these dietary parameters [231].

#### 8.2.5. Poultry

Incorporation of *C. sativa* seed meal into poultry diets benefits from the provisioning of energy and protein, increased health-promoting PUFAs and tocopherol content of meat and liver, improved antioxidant activity and lipid stability, and increased market value of the meat and eggs [189,232]. Unlike other livestock animals, chickens appear somewhat less susceptible to the taste of *C. sativa* seed meal and generally do not show reduced feeding efficiency or weight gain when provided in moderation. For example, the addition of 2.5%, 5%, and 10% *C. sativa* seed meal to a basal corn–soybean diet had little effect on weight gain and feed efficiency [233]. However, *C. sativa* seed meal resulted in significant increases in a-linolenic acid and n-3 PUFAs in white and dark meat and tissues [233]. Inclusion of 10% *C. sativa* seed meal increased egg production and increased α-linolenic acid and total n-3 PUFAs in egg yolks, compared with a corn–soybean-based diet, but reduced crude protein digestibility [234]. Addition of 8%, 16%, and 24% *C. sativa* seed meal resulted in linear increases in α-linolenic acid content in various tissues including liver, breast, and thigh, and in the proportion of n-3 PUFAs in liver and brain [235]. The supplementation of cold-pressed *C. sativa* seed cake of broiler chicken diets with or without multienzyme supplementation to improve digestibility was found to improve the energy and amino acid content of the diets [236].

Related studies tested the effects of oil supplements on poultry diets. For example, the addition of 6% of *C. sativa* oil to feed rations for broiler chickens reduced the cholesterol content in the blood plasma and increased omega-3 PUFA content in the breast, without producing an unpleasant flavor [237,238]. The addition of *C. sativa* oil to broiler chicken diets resulted in increased α-linolenic acid content in muscle and abdominal fat to a greater extent than diets supplemented with either soybean or rapeseed oil [237]. Other studies showed that *C. sativa* oil and seed meal in broiler chicken diets improved the percentage of PUFAs, particularly omega-3, and lowered the percentage of MUFAs, such as oleic acid, in the lipids of the breast muscles [239].

In addition to chicken, a variety of other poultry species were evaluated for *C. sativa* seed meal use. As with chicken diets, the inclusion of 15% to 20% *C. sativa* seed meal in duck diets resulted in significant increases in α-linolenic acid and total omega-3 PUFA in breast and leg muscles [240]. The addition of up to 5% *C. sativa* seed meal to the diets of young turkeys showed similar weight gain and feed conversion as vegetable oil and showed no negative effects [241]. However, the addition of 5% and 10% *C. sativa* cake to turkey diets caused growth depression and reduction of feed intake [242,243]. In contrast, no negative effects of weight gain or feed intake were observed when 5%, 10%, or 15% *C. sativa* seed meal was added to quail diets [244].

#### 8.2.6. Fish

*C. sativa* oil and seed meal can serve as good replacements for fish oil and fish meal in fish feed. Diets of 5% *C. sativa* seed meal are acceptable substitutes to reduce reliance on fish meal [245]. Substitution of *C. sativa* oil in fish feed improved the total lipid content in salmon (*Salmo salar* L.) and Atlantic cod (*Gadus morhua* L.) without negatively affecting the sensory quality of fish fillets [246]. *C. sativa* seed oil can be used as up to 100% replacement for fish oil without negative growth effects for Atlantic salmon raised in freshwater [245]. However, while replacing fish oil with *C. sativa* oil at ~80% had no effect on growth performance, PUFA content in the liver and muscle was reduced in Atlantic cod [247]. In another study with Atlantic cod, feeding *C. sativa* seed meal up to 24% of the diet did not affect growth in one experiment, while in a separate experiment, *C. sativa* seed diet greater than 30% resulted in depressed feed intake and growth due to reduced palatability [248]. Replacement of fish oil with oil from *C. sativa* genetically modified to express eicosapentaenoate (EPA; 20:5 n-3) and docosahexaenoate (DHA; 22:6 n-3) [249], in a feeding trial of European sea bass (*Dicentrarchus labrax*), showed no change in feed intake or growth [250]. However, the resulting fillets showed enhanced omega-3 PUFA content, indicating that *C. sativa* seed oil can effectively replace fish oil for the production of European sea bass without negative impacts on feed efficiency or growth rates [250]. *C. sativa* seed or seed meal of at least 10% can be used for feeding rainbow trout (*Oncorhynchus mykiss*) without reductions in feed conversion or weight gain [251].

### 8.3. Biodiesel/Renewable Diesel Fuel

One of the most important uses for *C. sativa* oil is the production of various biofuels. As with other oilseeds, the fatty acid profile of *C. sativa* oil varies depending upon the environmental conditions under which it is grown, thus resulting in variations in physical and chemical properties of the biodiesel produced. The major properties used to evaluate the quality and performance of biodiesel include cloud point (CP), cold-flow characteristics or viscosity, cetane number (CN), and oxidative stability [2,26]. Unformulated *C. sativa* B100 fails to meet the American Society for Testing and Materials (ASTM) standards for CP and oxidative stability due to exceptionally high (48–50%) polyunsaturated fatty acid content, but does meets the standard criteria for CN. However, with the addition of antioxidants, unformulated B100 can meet current ASTM standards while maintaining production costs at a level comparable with other biodiesel feedstocks [165,252]. Biodiesel produced from *C. sativa* oil generally resembles biodiesel from Canola oil (*B. napus*) [25]. The kinematic viscosity of methyl esters obtained from *C. sativa* varies from 2.9–6.4 mm^2^ s^−^^1^ at 40 °C, which lies within the acceptable 1.9–6.0 mm^2^ s^−^^1^ ASTM D651, and 3.5–5.0 mm^2^ s^−^^1^ EN14214 standard values [25,26,165,166,253,254,255]. The CN for *C. sativa* methyl ester ranged from 49.26 to 51.17, a value higher than ASTM biodiesel standards, resulting in good ignition quality of fuel. Furthermore, the pour point of *C. sativa* methyl ester was typically between −11 °C and −8 °C, suggesting a good quality fuel under cold season temperatures. Fuel consumption and vehicle operation from *C. sativa* and Canola methyl esters are similar [25]. In addition to traditional biodiesel, *C. sativa* can be used to produce hydrogenation-derived renewable diesel (HDRD) or green diesel, which generally has chemical properties more similar to that of petroleum diesel and improved cold-flow characteristics [256], yet is more sustainable than fossil diesel based upon emission parameters [257]. Life cycle analysis of *C. sativa* for biodiesel production showed that it produces lower greenhouse gas emissions than soybean- or Canola-derived biodiesel through reduced impacts on land-use changes [258].

### 8.4. Hydro-Processed Renewable Jet (HRJ) Fuel

Renewable jet fuel production from *C. sativa* oil follows a two-step standard process: (1) initial hydrodeoxygenation or hydrotreatment and (2) selective cracking or hydrocracking and isomerization, followed by product separation and formulation. The linear alkanes produced during step one can be used in renewable diesel mixtures [2,166]. Compared with JP-8 (typical jet fuel), *C. sativa* HRJ fuel had superior thermal-oxidative stability [259]. Engine tests with *C. sativa* fuel showed no obvious anomalies with engine operation, with the added advantage of lower carbon monoxide emissions than JP-8. However, *C. sativa* HRJ fuel contains elastomer seal swelling capability, which might cause fuel leaks in aircrafts, but this was lower than that of JP-8 [259]. *C. sativa* HRJ fuels have similar properties to conventional fuels used in turbine engines [259,260,261]. In 2009, the U.S. Air Force (USAF) tested the potential of *C. sativa* HRJ fuels as a replacement for jet fuels, with successful tests performed in fighter jets, private jets, and commercial airlines using a blend of JP-8 and *C. sativa*-based jet fuels [2,262]. *C. sativa*-based jet fuel with blending had a 75–80% lower carbon footprint compared with conventional fuel [263]. Overall, these results confirm the ability of renewable fuels derived from *C. sativa* to burn cleaner than conventional fossil fuel resources, while also removing carbon from the atmosphere during their production, resulting in overall lower carbon emissions. However, techno-economic analysis reveals that considerable investments in land area would be needed to grow *C. sativa* at large scale for economic viability [264].

### 8.5. Fast Pyrolysis Bio-Oil

In addition to the use of *C. sativa* for biodiesel and renewable diesel, the energetic value of the seed meal and lignocellulosic biomass was also considered. Thermochemical fast pyrolysis of the seed meal after oil extraction contains significant energetic value (29 MJ kg^−^^1^) when compared with whole seeds (34.7 MJ kg^−^^1^) and can provide additional volumes of high-energy, high-carbon liquid fuel intermediates for the production of renewable biofuels and jet fuel [265]. *C. sativa* residual biomass is comprised of 36.3–41.6% cellulose, 23.2–24.9% hemicellulose, and 25.0–26.3% lignin, and can be converted to bio-oil using thermal or catalytic fast pyrolysis [266]. However, the energetic value of *C. sativa* straw (15.1–15.5 MJ kg^−^^1^) or straw pellets (16.6–17.1 MJ kg^−^^1^) is lower than that of seeds or seed meal, but can produce gas, solid, and liquid fuel components with reduced nitrogen and sulfur contents relative to other woody or herbaceous feedstocks tested [267]. *C. sativa* straw was also used for ethanol production with an estimated total energy yield of 54.3 MJ L^−^^1^ ethanol [268]. Agronomic management can improve the overall energy potential of *C. sativa* biomass. For example, high rates of nitrogen and sulfur fertilization can increase the energy output of *C. sativa* seed and residual biomass by up to 186% and 155%, respectively [269]. The net energy efficiency and economic value of total *C. sativa* biomass (straw and seed) was higher than that of crambe (*Crambe abyssinica*) [270]. Thus, *C. sativa* biomass utilization, as part of an integrated biorefinery strategy where renewable diesel or jet fuel and thermal or catalytic fast pyrolysis bio-oil production are co-localized, can improve the overall energetic value of this bioenergy resource.

### 8.6. Industrial Applications

*C. sativa* oil has great potential for bio-based industrial applications. The high quantity of unsaturated fatty acids (~90%) present in *C. sativa* oil [6], enables rapid drying, and is useful for making dermatological products, cosmetics (lotions and soaps), polymers, paints, and varnishes [22,27,271]. A key limitation of using *C. sativa* oil for industrial application is its high content of PUFA, which promotes the oxidation of oils upon exposure to high temperatures [272,273]. *C. sativa* oil can be epoxidized and used for making adhesives, coatings, lubricants, and alkyd resins [274,275]. *C. sativa* meal has great potential for use in the paper industry [276]. The addition of *C. sativa* meal to recycled newspaper produced sustainable and bio-degradable green composite sheets and fibers [276]. Other applications include bio-herbicides [277], soil fungicides [278], and bio-oils [265]. *C. sativa* oil contains glucosinolates, which can be used to produce ionic thiocyanates that can serve as effective bio-herbicides against redroot pigweed (*Amaranthus retroflexus* L.) and wild oat (*Avena fatua* L.) [277]. *C. sativa* glucosinolate content ranged 9–31.4 µmol g^−^^1^ in seed [279,280], 14.5–23.4 µmol g^−^^1^ in seed meal [280], and 8.6–30.5 µmol g^−^^1^ in oil [281] depending upon the genotype and environmental growth conditions.

### 8.7. Biomedical Uses

In addition to the beneficial use of *C. sativa* seed meal for improving the quality of other foods such as meat and dairy products, the consumption of dietary oils, such as the oil from *C. sativa*, which is rich in essential omega-3 fatty acids (e.g., α-linolenic acid), was associated with reduced risks of coronary heart disease and inflammatory diseases [282,283]. Supplementation of the human diet with *C. sativa* oil can also reduce serum cholesterol in hypercholesterolemic subjects [179]. In addition, the oil contains high nutritional value due to the presence of tocopherols, phytosterols, and carotenoids [284,285].

Leaves of *C. sativa* contain flavonols such as quercetin [286]. Methanolic and ethanolic extracts of *C. sativa* seeds show significant antibacterial and antifungal activities and potential as natural preservatives [287]. Additional therapeutic properties are associated with phenolic compounds in *C. sativa* seeds and oil, such as chlorogenic, caffeic, sinapinic, and phytic acids [288]. However, as mentioned earlier, *C. sativa* seeds contain glucosinolates, which have antinutritive properties [201].

The ethanolic and methanolic extracts of defatted *C. sativa* seeds were shown to reverse short-term memory impairment and reduce anxiety and depression-like behaviors in a Swiss mouse model for irritable bowel syndrome exposed to various stress tests [289]. Furthermore, these extracts decreased superoxide dismutase, but increased glutathione peroxidase activity in brain and bowel tissues as well as potentially increasing lipid peroxidation in the bowel. The authors concluded that seed extracts could improve performance and mood while exhibiting antioxidant capacity in both brain and bowel tissues [289]. In a related study, the administration of cold-pressed *C. sativa* oil was shown to ameliorate impairment of short-term memory, anxiety, and depression in a Swiss mouse zymosan-induced model for irritable bowel syndrome exposed to various stress tests [290]. Furthermore, *C. sativa* oil treatment resulted in increases in superoxide dismutase and glutathione peroxidase activity in brain and bowel tissues, resulting in decreased malondialdehyde levels, a lipid peroxidation marker, in these tissues [290].

## 9. Strategic Innovations for Climate-Resilient *C. sativa*

The hotter and drier conditions brought about by climate change will require novel strategies for improving the heat and drought durability of *C. sativa* in the future. Recent research suggests that *C. sativa* is able to maintain a higher shoot/root ratio under water-deficit stress and is more drought tolerant than Canola (*B. napus*) [291]. Comparisons of *C. sativa* and Canola under deficit irrigation regimes suggest that *C. sativa* performed better and gave higher seed yields than Canola under field conditions [292]. To assess the relative tolerance to water-deficit and salinity stress, screening assays of different genotypes at germination were developed. Such germination screens suggested that seedlings of *C. sativa* cv. Calena exhibited germination up to 150 mM NaCl and thus might be suitable for cultivation in saline soils [293], but additional research is required to confirm this suggestion. Comparison of two different genotypes of *C. sativa* showed that increased water-deficit stress tolerance was associated with differences in stomatal conductance and increased accumulation of osmoprotectants such as proline, sugars, amino acids, and soluble proteins [294]. Screening of winter and spring genotypes revealed differences in germination under artificial osmotic stress imposed by different concentrations of polyethylene glycol (PEG) [295]. Application of sodium selenite (Na_2_SeO_3_) via seed priming or foliar application was shown to increase drought tolerance in *C. sativa* (and Canola) via the accumulation of osmoprotectants and antioxidants, and to improve crop yields [296,297]. Fertilization of *C. sativa* with thiourea at the seed filling stage improved heat tolerance [298]. While such selection or treatment efforts carry a certain degree of practical utility, more direct advanced breeding and biotechnological innovations will be needed to dramatically improve the yield potential and climate resiliency of *C. sativa*.

### 9.1. Molecular Breeding Approaches

Characterizing the genetic diversity and population structure of available genotypes is often the first step towards developing breeding programs for improving key traits such as yield potential, oil content, resistance to biotic and abiotic stresses, and adaptability to diverse environments. Sequencing complete allohexaploid *C. sativa* transcriptomes and genomes was a key first step for advanced molecular breeding efforts [34,53,299,300]. Such information is critically important for future gene identification using genomic selection, marker-assisted selection, and genome-wide associations studies (GWAS) to guide future *C. sativa* breeding programs. For example, a recombinant inbred population from two *C. sativa* accessions (Suneson and Pryzeth) with contrasting traits, especially seed size and oil content, was developed and used to assess phenotypic differences under two environmental conditions (dryland and irrigated) [301]. Using 189 lines from this population, a genetic map was created containing 2376 single nucleotide polymorphisms (SNPs) to identify quantitative trait loci (QTLs) associated with oil content, seed size, pod size, and seed number per pod as a first step towards isolating genes that control for seed development and oil accumulation [301]. In another example, a total of 213 spring *C. sativa* accessions were collected and genotyped to assess the genetic diversity available for future breeding efforts [302]. A total of 6192 SNPs were identified using genotyping-by-sequencing technology and used to reveal two distinct populations of *C. sativa* arising from Germany and eastern Europe [302].

Various screening efforts for wildtype and mutagenized *C. sativa* were used to identify lines with desirable traits of interest. For example, analysis of *C. sativa* seedlings from 10 different cultivars using germination screens under a gradient of NaCl conditions found that 100 mM NaCl was optimal for differentiating the phenotypic performances of seedlings [303]. Screening of a spring panel of 211 *C. sativa* accessions followed by GWAS revealed a total of 17 significant trait-associated SNPs for germination rates and dry weight with potential roles in root development through mediation of phosphate metabolism, signaling, and cell membrane activities [303]. In addition, mutational breeding using gamma-ray irradiation was successfully used to alter the fatty acid profile in *C. sativa*, resulting in the identification of M_2_ mutants with increased α linolenic acid content or decreased erucic acid content [304]. Screening of ethyl methanesulfonate (EMS) mutant populations resulted in the isolation of a *C. sativa* mutant with reduced seed coat mucilage, which could potentially improve the flow characteristics of the oil and reduce the washing requirements of the resultant biodiesel [305].

### 9.2. Improving Productivity Using Rhizosphere Microbes

In order to reduce reliance on fossil fuel-based fertilizers, interest into the use of plant growth-promoting rhizobacteria has increased in recent years. Early research indicated that the presence of nitrogen-fixing bacteria was necessary to stimulate root growth in flax (*Linum usitatissimum* L.) through aqueous washing of *C. sativa* leaves [306]. More recently, inoculation of *C. sativa* with a consortium of rhizosphere soil bacteria containing *Bacillus* species resulted in approximately a threefold increase in seed yield [307]. Similar enhancement in *C. sativa* winter hardiness and seed productivity were observed when *C. sativa* seeds were inoculated with nodule bacteria from crimson clover (*Trifolium incarnatum* L.) roots before sowing [308].

### 9.3. Improving Quality Traits

To complement molecular breeding and rhizosphere manipulation approaches, advances in the genetic transformation of *C. sativa* were developed [9,10,12,13]. The stable introduction of genes into *C. sativa* was used to modify or improve a wide range of agronomic traits with a focus on the manipulation of seed oil yield potential and characteristics [11]. For example, *C. sativa* was genetically modified using a set of heterologous genes to express eicosapentaenoate (EPA; 20:5 n-3) and docosahexaenoate (DHA; 22:6 n-3) as a replacement for fish oil [249]. Improving the oil content and seed yield in *C. sativa* by co-expressing *A. thaliana* diacylglycerol acyltransferase 1 (DGAT1) and yeast cytosolic glycerol-3 phosphate dehydrogenase (GPD1) genes under the control of seed-specific promoters resulted in up to 13% higher seed oil content and up to 52% higher seed mass compared with wild-type plants [309].

### 9.4. Improving Stress Tolerance Traits

Soil amendments containing PGPR (*Pseudomonas migulae* 8R6) that produce 1-aminocyclopropane-1-carboxylate deaminase (ACC deaminase) increased seed production in *C. sativa* by 30–50% under salinity stress by reducing stress ethylene [310]. Furthermore, plants expressing the bacterial version of the ACC deaminase (acdS) gene showed higher seed production, better seed quality, and higher levels of seed oil production under salinity stress than control lines [310]. The observed improvements in salinity tolerance were attributed to changes in the gene expression of multiple plant signaling pathway components and the modulation of genes involved in the production of reactive oxygen species (ROS) scavenging and signaling [311].

Many engineering strategies targeting improvement of abiotic stress tolerance begin with the characterization of stress-responsive genes. For *C. sativa*, stress-responsive gene discovery was performed by subjecting 15-day-old plants to 3 or 17 days of water-deficit stress and rehydration recovery for 3 days to identify hundreds of differentially expressed genes in diverse metabolic and signaling pathways based upon their relative, steady-state transcript abundance patterns [312]. Gene expression changes in *C. sativa* roots and vegetative tissues in response to salinity stress revealed hundreds of changes in relative, steady-state transcript abundance, the relative proportion of which could be assigned to each of the three *C. sativa* subgenomes [313]. Ectopic overexpression of a MYB96 transcription factor from *A. thaliana* in *C. sativa* resulted in improved water-deficit stress tolerance, due to the activation of genes involved in epicuticular wax biosynthesis [314]. Similarly, the ectopic overexpression of the CsMYB96A gene resulted in increased water-deficit stress tolerance due to the increased expression of epicuticular wax biosynthetic genes [315]. Such an approach might be useful for growing *C. sativa* on semi-arid and arid lands; however, field trials would be necessary to assess the efficacy of these modifications.

### 9.5. Genome-Editing Approaches

To complement molecular breeding approaches, advances in genetic transformation and genome editing using clustered regularly interspaced short palindromic repeats (CRISPR)/CRISPR-associated protein (CRISPR/Cas) are used to improve the oil contents and fatty acid profiles in oilseed crops, including *C. sativa* [316,317,318]. An early demonstration of this technology in *C. sativa* was the simultaneous mutation of the diacylglycerol acyltransferase (DGAT1) and phospholipid:diacylglycerol acyltransferase (PDAT1) genes, which resulted in decreased oil content and altered fatty acid composition [319]. CRISPR/Cas9 disruption of all three homoeologous fatty acid desaturase 2 (FAD2) genes in *C. sativa* resulted in a substantial increase in oleic acid content (15% to >50%) with associated decreases in linoleic (~16% to <4%) and α-linolenic acid (~35% to <10%) content in *C. sativa* seeds [16]. In a related study, disruption of all three pairs of FAD2 homeologs in *C. sativa* resulted in an 80% enhancement in MUFA content in seeds, but this resulted in severely stunted plants [319]. However, if only two homeologous gene pairs were knocked out, up to a 60% increase in seed MUFAs was obtained and the plants showed normal phenotypes [319]. This alteration in the fatty acid profile resulted in a healthier oil profile while also improving the oxidative stability of the oil, which improves its utility for biodiesel production [16]. A related study used CRISPR/Cas9 to knock out all three FAD2 homeologs in the allohexaploid *C. sativa*, resulting in increased accumulation of oleic acid in the seed oil [15]. CRISPR/Cas9-mediated knockout mutagenesis of all three fatty acid elongase 1 (FAE1) genes reduced the total content of C20-C24 very long-chain fatty acids (VLCFAs), which normally constitute 22% of fatty acids in wild-type plants, to less than 2% of total fatty acids, with a corresponding increase in desirable C18 unsaturated fatty acids for dietary or fuel uses [14]. CRISPR/Cas9 gene editing was also used to disrupt the three homeologous genes encoding CRUCIFERIN C, which resulted in an alteration in the relative abundance of other cruciferin isoforms, but did not alter total seed protein content [320]. However, the overall amino acid composition of the seed was altered along with an increase in the relative abundance of all saturated fatty acids. Other attractive targets of genome editing to improve production traits include strategies to reduce silicle shattering and to prevent uneven seed maturation.

## 10. Conclusions and Future Directions

*C. sativa* is a highly attractive oilseed due to its low input requirements and costs and adaptability to diverse environments and soil conditions, including abandoned, marginalized, and semi-arid and arid regions. While genetic engineering and genome-editing approaches have yielded great improvements in its oil content and fatty acid profiles, *C. sativa* can also be outcrossed with related species [103], raising the possibility of targeted breeding programs to improve its yield and trait diversity. Improvements in agronomic management and commercial investments are also needed to reduce production costs and inputs and improve its overall seed yield and seed oil content relative to competing oilseeds [1,258]. In addition, improved valorization of the *C. sativa* value chain, from seed processing, oil extraction and refinement methods [321] to its co-products and lignocellulosic crop residues, is needed to better understand its integrated economic value [322] within the framework of the biorefinery concept and the bio-based economy for *C. sativa* [270,323]. More detailed examination of phenotypes is also necessary to discover natural variation in quality traits, such as the low erucic acid content of its oil [161], and production traits, such as improved heat and drought tolerance [324]. Lastly, increasing market demands for bio-based products for the renewable fuels market and improvements in value-chain cost efficiencies will likely increase future demands for this versatile crop, with great potential for agricultural production systems of the future that will increasingly rely on more climate-resilient crops such as *C. sativa*.

## Figures and Tables

**Table 2:** Fatty acid profiles of *C. sativa* seed oil reported by various researchers. Bold font (bottom row) indicates mean values of fatty acid profile in *C. sativa* from the different reports. Dashes indicate missing data.

Concentration of Major Fatty Acids (%)										Reference
C16:0	C18:0	C18:1	C18:2	C18:3	C20:1	C22:1	SFA	MUFA	PUFA	
-	-	-	21–23	27–29	-	-	11–12	35–36	51–52	[75]
-	-	14–16	15–23	31–40	12–15	-	-	-	-	[2]
5.3–5.6	2.2–2.7	14.7–16.5	12.9–16.3	35	15	3	12	34	54	[159,160]
-	-	-	-	30–43	11–19	<3	-	-	-	[6]
5.4	2.4	14.3	20.6	36.9	13	2.2	-	-	-	[3]
6.29	2.73	16.5	17.7	32.5	15.6	3.1	-	-	-	[64]
5.16	2.68	15.2	17.9	34.6	15.1	2.6	8.6	33.0	54.1	[26]
5.68	-	13.9	16.6	35.1	14.3	3.0	10.1	33.5	55.9	[164]
-	-	9.1–22.1	15.2–27.1	22.8–38.4	11.6–18.2	-	-	-	-	[158]
5.5	2.4	14.4	19.1	33.5	15	3.1	-	-	-	[165]
7	2.5	6.9	14.5	41	10.9	3.5	-	-	-	[157]
6.8	2.7	18.6	19.6	32.6	12.4	2.3	-	-	-	[166]
5.1	2.4	17.6	18.7	28.6	11.9	4.2	-	-	-	[167]
5.4	2.6	14.3	14.3	38.4	16.8	2.9	-	-	-	[25]
5.7	3.4	15.0	18.5	34.7	12.7	3.2	-	-	-	[168]
4.6–5.2	2.2–2.5	12.8–14.7	16.3–17.2	36.2–39.4	14.0–15.5	2.5–3.1	8.7–8.8	-	-	[39]
5.5–9.5	-	9.1–17.1	16.1–28.6	23.5–36.2	10.5–16.4	<2	-	-	-	[161]
6.2	2.6	16.2	17.5	37.3	13.1	2.3	9.7	31.7	57.7	[71]
**5.8**	**2.6**	**14.7**	**18.3**	**34.4**	**14.0**	**2.9**	**10.1**	**33.5**	**54.6**	

## Data Availability

Not applicable.
